# Dynamic ploidy changes drive fluconazole resistance in human cryptococcal meningitis

**DOI:** 10.1172/JCI124516

**Published:** 2019-01-28

**Authors:** Neil R.H. Stone, Johanna Rhodes, Matthew C. Fisher, Sayoki Mfinanga, Sokoine Kivuyo, Joan Rugemalila, Ella Shtifman Segal, Leor Needleman, Síle F. Molloy, June Kwon-Chung, Thomas S. Harrison, William Hope, Judith Berman, Tihana Bicanic

**Affiliations:** 1 Centre for Global Health, Institute for Infection and Immunity, St. George’s, University of London, United Kingdom.; 2 MRC Centre for Global Infectious Disease Analysis, School of Public Health, Imperial College London, London, United Kingdom.; 3 National Institute of Medical Research, Dar es Salaam, Tanzania.; 4 Liverpool School of Tropical Medicine, United Kingdom.; 5 Muhimbili National Hospital, Dar es Salaam, Tanzania.; 6 School of Molecular Cell Biology and Biotechnology, Tel Aviv University, Israel.; 7 National Institutes of Health, Bethesda, Maryland, USA.; 8 Institute of Translational Medicine, University of Liverpool, United Kingdom.

**Keywords:** AIDS/HIV, Infectious disease, Drug therapy, Fungal infections

## Abstract

**BACKGROUND.** Cryptococcal meningitis (CM) causes an estimated 180,000 deaths annually, predominantly in sub-Saharan Africa, where most patients receive fluconazole (FLC) monotherapy. While relapse after FLC monotherapy with resistant strains is frequently observed, the mechanisms and impact of emergence of FLC resistance in human CM are poorly understood. Heteroresistance (HetR) — a resistant subpopulation within a susceptible strain — is a recently described phenomenon in *Cryptococcus neoformans* (*Cn*) and *Cryptococcus gattii* (*Cg*), the significance of which has not previously been studied in humans.

**METHODS.** A cohort of 20 patients with HIV-associated CM in Tanzania was prospectively observed during therapy with either FLC monotherapy or in combination with flucytosine (5FC). Total and resistant subpopulations of *Cryptococcus spp*. were quantified directly from patient cerebrospinal fluid (CSF). Stored isolates underwent whole genome sequencing and phenotypic characterization.

**RESULTS.** Heteroresistance was detectable in *Cryptococcus spp*. in the CSF of all patients at baseline (i.e., prior to initiation of therapy). During FLC monotherapy, the proportion of resistant colonies in the CSF increased during the first 2 weeks of treatment. In contrast, no resistant subpopulation was detectable in CSF by day 14 in those receiving a combination of FLC and 5FC. Genomic analysis revealed high rates of aneuploidy in heteroresistant colonies as well as in relapse isolates, with chromosome 1 (Chr1) disomy predominating. This is apparently due to the presence on Chr1 of *ERG11*, which is the FLC drug target, and *AFR1,* which encodes a drug efflux pump. In vitro efflux levels positively correlated with the level of heteroresistance.

**CONCLUSION.** Our findings demonstrate for what we believe is the first time the presence and emergence of aneuploidy-driven FLC heteroresistance in human CM, association of efflux levels with heteroresistance, and the successful suppression of heteroresistance with 5FC/FLC combination therapy.

**FUNDING.** This work was supported by the Wellcome Trust Strategic Award for Medical Mycology and Fungal Immunology 097377/Z/11/Z and the Daniel Turnberg Travel Fellowship.

## Introduction

Cryptococcal meningitis (CM) is a common and severe opportunistic infection primarily affecting patients with advanced HIV/AIDS caused by the basidiomycete yeasts *Cryptococcus neoformans* (*Cn*) or less frequently *Cryptococcus gattii* (1). CM mortality is particularly high in resource-limited settings where mortality can exceed 50% even with antifungal treatment (2, 3). CM is invariably fatal without treatment (4). Recent estimates place the annual death toll from CM approaching 200,000 deaths per year, predominantly in sub-Saharan Africa (5). The gold standard of treatment for CM in HIV-infected patients is a 2-week induction regimen of amphotericin B deoxycholate (AmB) combined with flucytosine (5FC), followed by a consolidation and then maintenance phase with oral fluconazole (FLC) (6). In many countries, however, and particularly those in sub-Saharan Africa, FLC is the only available antifungal; therefore, FLC monotherapy is used for induction, consolidation, and long-term maintenance regimes (7). Even with relatively high doses of 1200 mg/day of FLC, CM treatment outcomes with FLC monotherapy as induction treatment are poor (2, 8).

The relationship between FLC resistance and CM treatment failure is not well understood. No clinical in vitro susceptibility breakpoints are available, because evidence for correlation between minimum inhibitory concentration (MIC) and outcome is sparse and conflicting (9, 10). Primary FLC resistance has not been considered as a major clinical problem because *Cn* generally displays low MICs to FLC in large epidemiological surveys (11). Nonetheless, clinical failure due to the recurrence of *C*. *neoformans* with high FLC MIC has been reported (12), and more recent data suggest FLC resistance (i.e., MIC > 8 mg/l) is as high as 10% in primary isolates and 24% in relapse isolates (13).

Heteroresistance to antifungal drugs is a recognized yet poorly understood subpopulation phenomenon observed in pathogenic fungi under drug pressure (14). As with bacteria such as *Staphylococcus aureus* (15) and *Mycobacterium tuberculosis* (16), studies of heteroresistance in fungi have been hampered by a lack of consistency in methodology and definitions (17, 18). Heteroresistance to FLC has been detected in clinical strains of *Candida glabrata,* although the mechanisms and clinical impact of this remain unknown (19). The phenomenon of heteroresistance to FLC in *C*. *neoformans* was first observed in vitro in 1999 (20, 21), and similarly in *C*. *gattii* (22), but was only recently linked to emergence of FLC resistance via aneuploidy both in vitro and in animal studies (23). However, until now, no data on FLC heteroresistance in human cryptococcal infection have been reported.

In vitro studies of *C*. *neoformans* found FLC heteroresistance to be intrinsic, that is, present in all strains regardless of prior drug exposure (24). Moreover, laboratory animal studies found that the size of the resistant subpopulation (the proportion of resistant cells as a percentage of the total) increases over time during FLC treatment (23). The predominant mechanism for this phenomenon appears to be aneuploidy, in particular transient duplication of chromosome 1 (Chr1), in heteroresistant colonies of *C*. *neoformans* (25). Two genes on Chr1 are known to play an important role in cryptococcal resistance to FLC: *ERG11*, which encodes the FLC target enzyme lanosterol-α-demethylase (26), and *AFR1*, an ABC-type efflux pump (27).

Here we examined the role of FLC heteroresistance in human cryptococcosis in a prospective observational study. This was done using cultures of serial samples of fresh ex vivo CSF on drug-free and drug-containing media in a clinical cohort of 20 HIV-infected patients receiving FLC-based induction therapy for CM in Tanzania, East Africa. We found that FLC heteroresistance is an intrinsic feature of clinical *Cn* isolates in HIV-infected patients at CM diagnosis, with a resistant subpopulation ranging from 0.01 % to 25% of the total (%HetR). We demonstrated that disomy, particularly of Chr1, was common in baseline heteroresistant isolates and correlated with phenotypic measures of resistance in vitro, including MIC and efflux pump activity. Importantly, the size of the heteroresistant subpopulation increased (elevated %HetR) within 2 weeks in patients receiving FLU monotherapy. This lead to aneuploidy-mediated clinical relapse with elevated MICs in 2 patients. Lastly, combination therapy with 5FC in addition to FLC was effective at suppressing amplification of the heteroresistant subpopulation in vivo, lending further support for oral combination therapies for African patients with CM.

## Results

**Figure 1:**
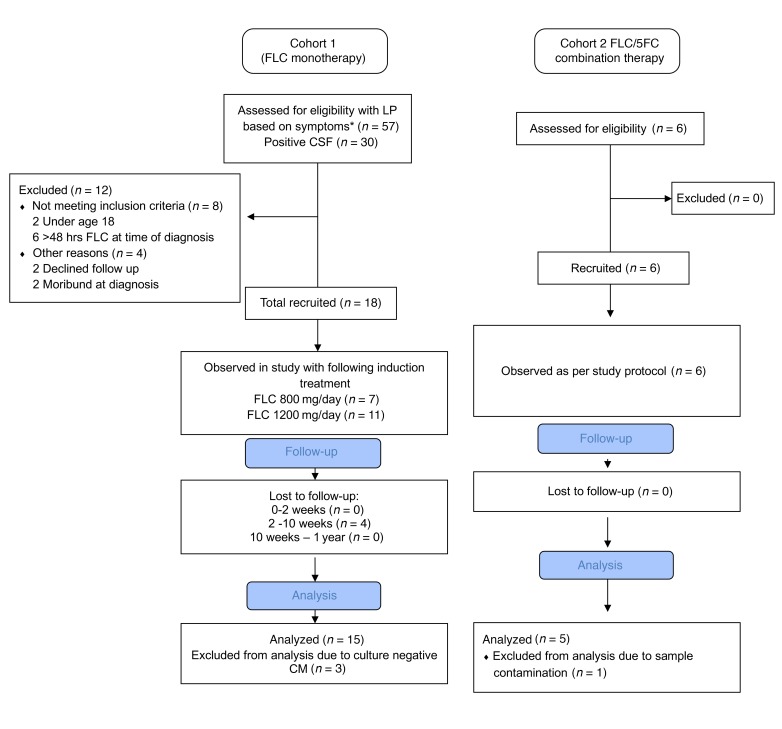
Flowchart of patients recruited to the clinical observational study. Patients in cohort 1 received induction treatment as per local treatment policy, either 800 mg or 1200 mg per day FLC for the first 2 weeks. Patients in cohort 2 had already been randomized to receive FLC/5FC combined as part of the ACTA trial and were approached to consent to be observed as part of this substudy. Asterisk indicates fever plus one or more of headache, confusion or reduced level of consciousness.

Following informed consent, 24 HIV-infected adult (age 18 or over) patients with a confirmed first episode of CM were prospectively recruited in Dar es Salaam, Tanzania (Figure 1). The patients received either FLC monotherapy (800–1200 mg/day) in line with local and national treatment policies or in combination with 5FC (100 mg/kg/d in 4 divided doses) as part of the ACTA trial (Supplemental Table 1; supplemental material available online with this article; https://doi.org/10.1172/JCI124516DS1). Serial clinical *Cryptococcus* isolates were obtained from 20 patients for phenotypic and genomic analyses (3 patients had culture-negative cryptococcosis and one strain was unsuitable for study due to sample contamination with environmental mold). One strain (2201) was subsequently found to be *C*. *gattii*. Patient characteristics at baseline are summarized in Supplemental Table 2. Baseline/pretreatment MICs of 17 of the strains were susceptible using Clinical and Laboratory Standards Institute (CLSI) breakpoints extrapolated from *Candida* species (≤ 8 mg/l) (11), with the other 3 falling into the “susceptible, dose-dependent” category of 16–24 mg/l. By 1-year follow-up of the study, 3 patients, all of whom had received FLC monotherapy, experienced clinical relapse. All-cause, 1-year mortality was 50% (Supplemental Table 2).

### Detection of heteroresistance in all clinical strains prior to initiation of fluconazole therapy.

Ex vivo direct testing of patient CSF was performed by inoculating CSF within 1 hour of clinical sample collection onto YPD agar supplemented with a range of FLC concentrations (8–64 mg/l). This identified a subpopulation of colonies able to grow on supra-MIC concentrations at baseline, prior to the patient receiving any FLC treatment. The growth on supra-MIC FLC concentrations was observed at a frequency ranging from 0.01% to 25% of the total CFUs per milliliter of CSF.

**Figure 2:**
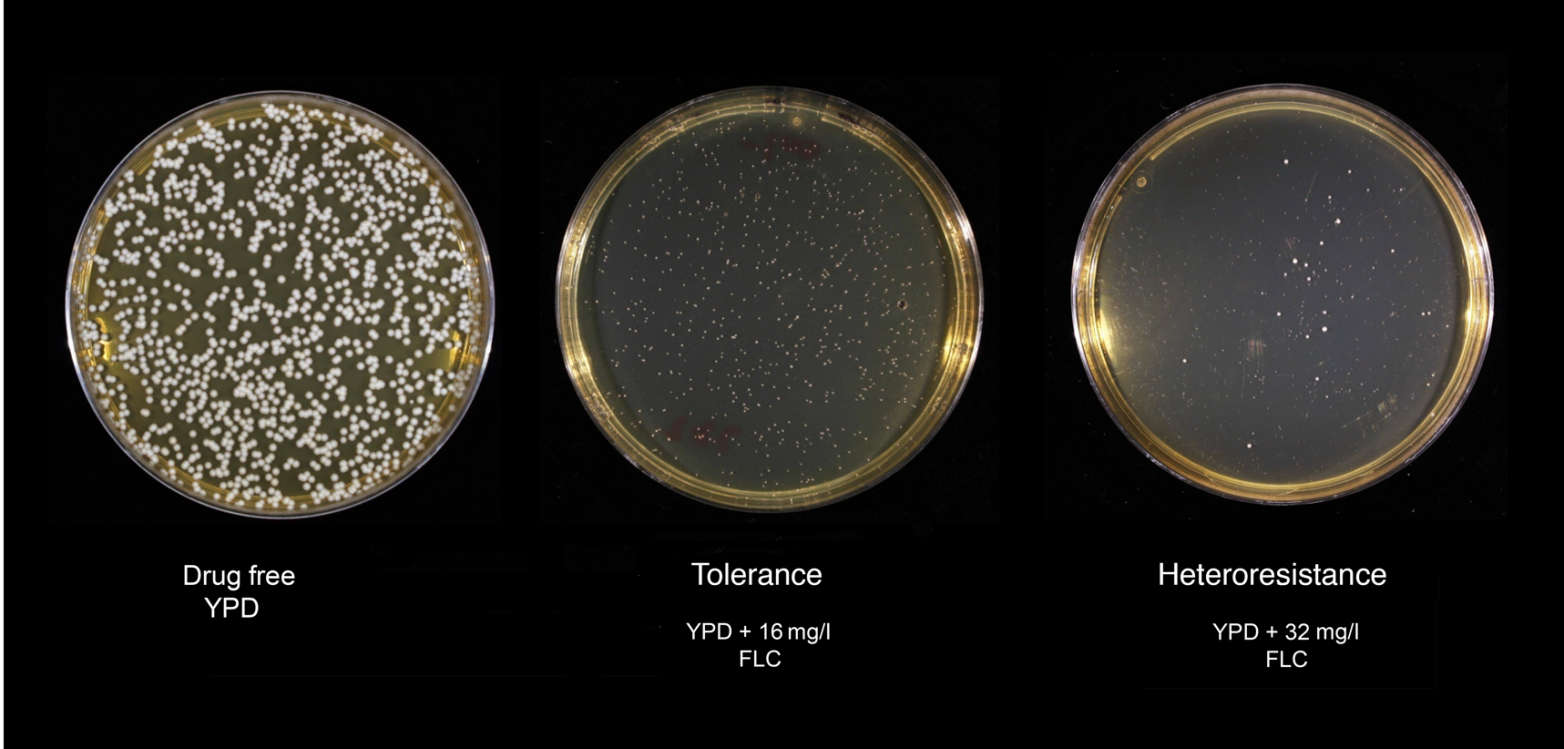
Phenotypes of tolerance and heteroresistance. *Cn* (10^6^) cells from the H99 reference strain showing colonies growing at the same quantity but smaller and more translucent at 16 mg/l. These colonies have the same MIC as the parent strain (1 mg/l) and are thought to represent tolerance. When plated on 32 mg/l FLC, a small percentage of opaque, medium-sized colonies appear, and have MIC of 256 mg/l, far higher than the parent strain. This represents true heteroresistance.

**Figure 3:**
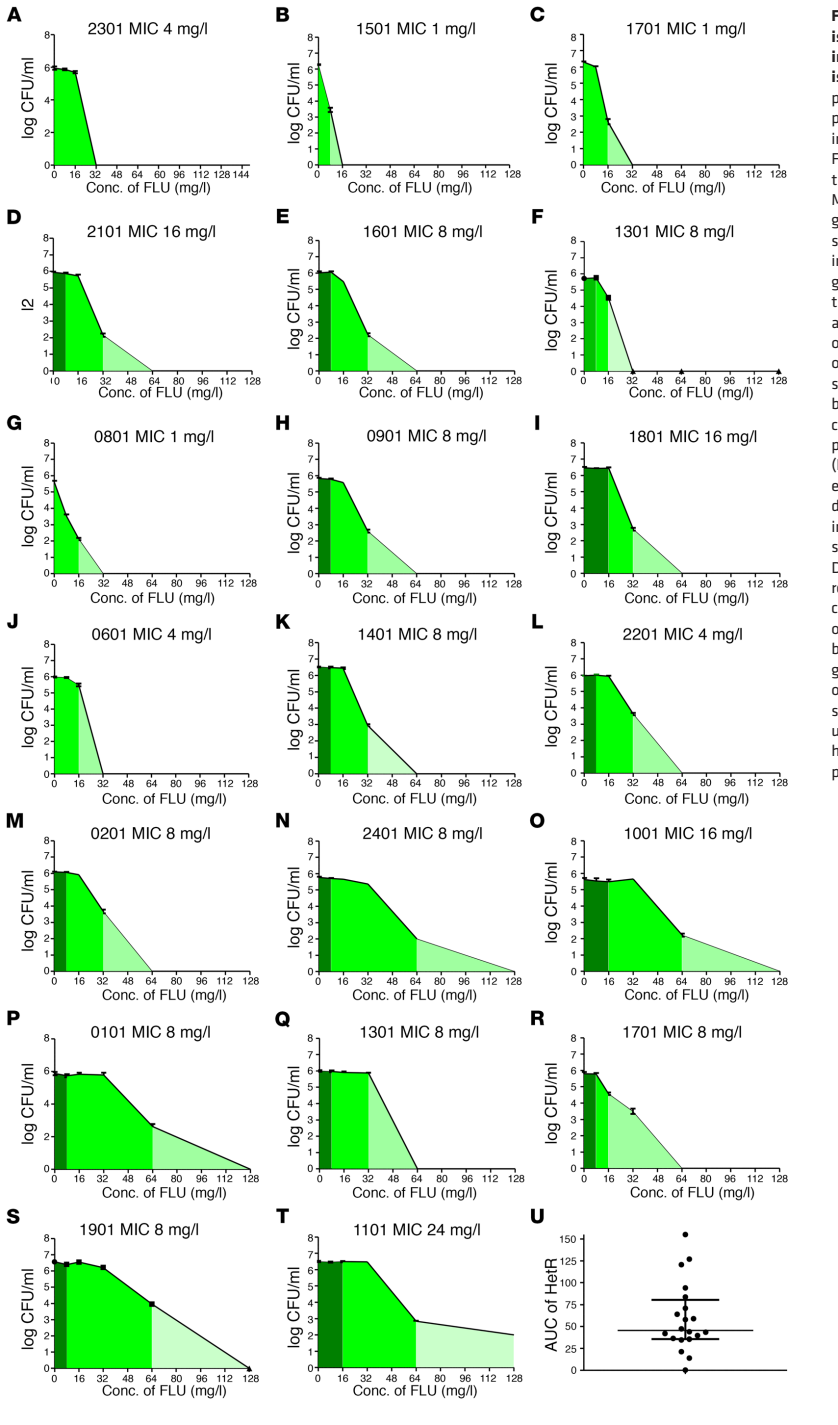
Heteroresistance is a continuous variable in clinical *C.* ***neoformans*isolates.** Population analysis profile (PAP) curves: CFUs per milliliter plotted against increasing concentrations of FLC. Dark green represents the susceptible population. Middle shade of green is the growth beyond the MIC of the strain, which grows poorly yet in a quantity similar to that grown on plain agar, and has the same MIC as the strain as a whole. The lightest shade of green is the subpopulation of colonies, which grows as a small percentage of the total but has an elevated MIC as compared with the dominant population, heteroresistance (HetR). (**A**–**T**) PAP curves for each of the 20 clinical isolates demonstrating heterogeneity in the AUC of HetR even for strains with similar MICs. Data points with error bars represent mean of quadruplicates ± SEM. (**U**) Distribution of heteroresistance (as defined by AUC of the subpopulation growing beyond the MIC, HetR) of the clinical isolates in the study, demonstrating a continuous distribution, rather than heteroresistance being a binary property of a strain.

Following a single passage on YPD agar, in vitro population analysis profiling (PAP) was used to measure the response to FLC of all stored baseline clinical isolates (strains ending in –01, Table 1) using a controlled starting inoculum of 1 × 10^6^ cells. All strains were able to grow on agar containing FLC concentrations above MIC, as determined by Etest. At lower concentrations of FLC (8–32 mg/l MIC, depending on the strain), similar numbers of colonies appeared. These colonies were smaller and more translucent than colonies grown on drug-free agar. These colonies had the same MICs as colonies growing on drug-free agar, suggesting that they most likely represent tolerance, a transient ability to survive, at slower growth rates, on supra-MIC drug concentrations (28). At higher FLC concentrations, a small percentage of colonies were able to grow in the presence of FLC in a strain-dependent manner. When tested independently, these colonies had a significantly elevated FLC MIC (≤ 4-fold) compared with the majority population, representing heteroresistance (Figure 2). PAP assay results were plotted as the log_10_ CFU/ml versus drug concentration and the AUC of the resistant subpopulation (i.e., the area under the curve of the PAP graph) was used as the parameter representing the heteroresistant subpopulation. The PAP curve can be split into 3 regions: (a) growth at concentrations below the MIC of the majority of the cells (susceptible); (b) visible but weak growth of the entire cell population on agar containing drug at concentrations beyond its MIC (tolerant growth); and (c) growth of a subpopulation of colonies at supra-MIC drug concentrations, which when retested have a higher MIC than the parent strain (heteroresistant cells) (Figure 3).

Individual PAP curves for each strain show inter-strain variability, including variability in heteroresistance (measured by PAP assay) among strains with the same MIC level (measured by Etest) (Figure 3B). Using the AUC of this heteroresistant subpopulation as a proxy for heteroresistance level, we found that heteroresistance is a continuous rather than a binary property. Furthermore, all but one of the clinical isolates analyzed in this study exhibited some degree of heteroresistance in vitro (Figure 3C). In vitro PAP assays reached results similar to the clinical assays of heteroresistance (i.e., percentage of colonies growing on drug vs drug-free media) performed directly from patient CSF. Although baseline percentage of HetR colonies was generally low, there was only a moderate, albeit statistically significant, relationship between the MIC and the level of heteroresistance (defined by the AUC of HetR) of a given clinical isolate (*r*^2^ 0.35, *P* = 0.005), suggesting that MIC may not be a reliable predictor of heteroresistance.

### Combination therapy with 5FC prevents amplification of fluconazole heteroresistance ex vivo.

**Figure 4:**
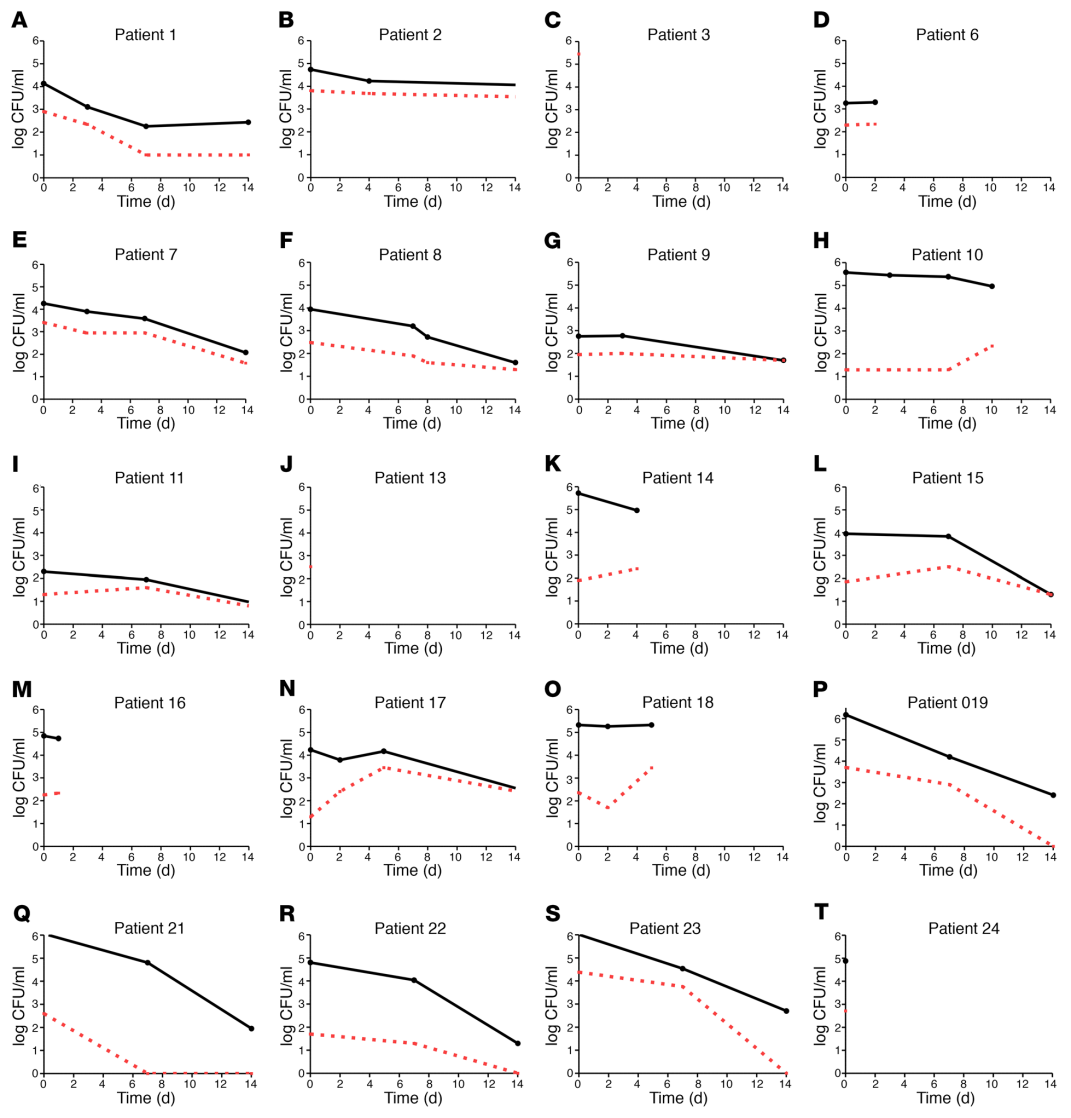
FLC monotherapy causes an increase in the resistant subpopulation in patient CSF, which can be prevented by the addition of 5FC. Fungal burden dynamics for each patient according to treatment received. The black line is the total population in CSF (log_10_ CFU/ml in CSF) whereas the red line is the subpopulation that grew on FLC-containing agar. (**A**–**O**) Patients receiving monotherapy, amplification of the resistant subpopulation is observed. (**P**–**T**) In the combination group, the subpopulation is undetectable by day 14 in all cases.

Prior to the start of the phase III Advancing Cryptococcal Treatment for Africa (ACTA) clinical trial (29) in Tanzania, patients received either FLC monotherapy at doses of either 800–1200 mg/day, standard practice per Tanzanian national treatment guidelines (30). After initiation of the ACTA trial, patients recruited to the trial and randomized to combination therapy of FLC at 1200 mg/day with 5FC at 100 mg/kg/day (4 divided doses of 25 mg/kg/d) as induction therapy were approached for inclusion in this substudy of heteroresistance. All patients received 400 mg FLC monotherapy (or 800 mg until ART initiation) for an additional 8 weeks and then 200 mg daily thereafter, in accordance with national guidelines. The dynamics of the total CSF fungal burden in CFU/ml, as well as the size of the subpopulation of cells able to grow on FLC-containing media over the course of FLC therapy, is shown for each individual patient in Figure 4.

**Figure 5:**
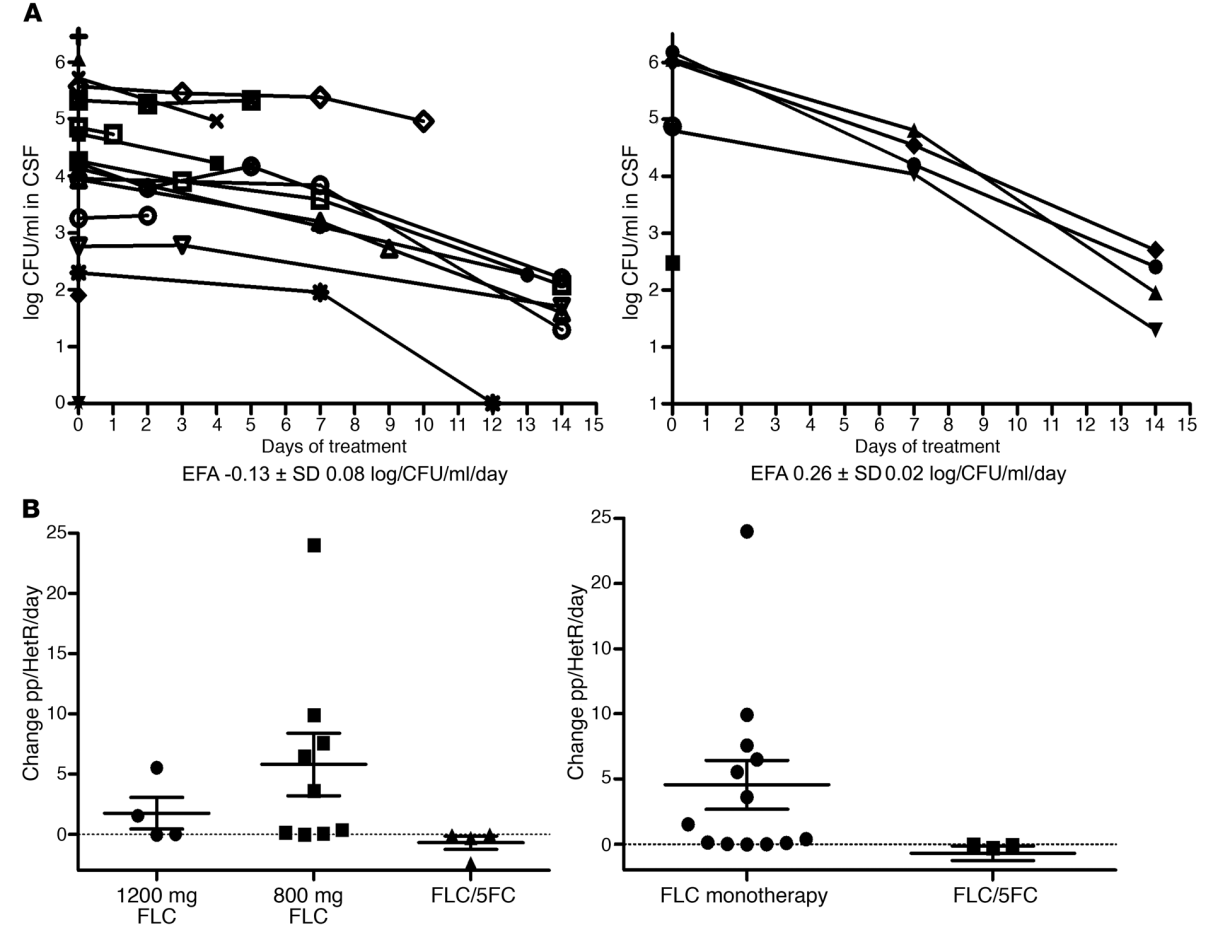
A combination of FLC and 5FC clears *Cryptococcus* faster from CSF, and also prevents the emergence of a FLC-resistant subpopulation. (**A**) EFA of monotherapy (*n* = 15) and combination treatment (*n* = 5), showing a faster rate of clearance in the combination arm (*P* = 0.02). (**B**) Mean rate of change of %HetR per day by treatment group was significantly different between the groups, with combination arm suppressing rather than amplifying the subpopulation (*P* = 0.019). Individual data points with SEM are displayed. Two-tailed Student’s *t* test, with *P* < 0.05 considered significant.

Early fungicidal activity (EFA), defined as the mean rate of decline in CSF log_10_ CFU/ml per day for each drug treatment group (Figure 5A) was consistent with prior cohorts (31). The combination of FLC/5FC had a significantly greater EFA compared with FLC monotherapy (mean + SD –0.11 ± 0.08, –0.13 ± 0.08 and –0.26 ± 0.02 log CFU/ml/d for FLC 800 mg, 1200 mg and combination therapy, respectively, *P* = 0.02 combination vs FLC, using the Student’s *t* test). In addition to the faster rate of clearance, there was a notable difference in the amplification of heteroresistance in the combination therapy versus monotherapy groups. In all 5 patients receiving FLC/5FC combination therapy, the heteroresistant subpopulation was undetectable by day 14. In contrast, in 11 of 13 patients (85%) receiving FLC monotherapy, within the first 7 days of treatment, the heteroresistance level increased: the proportion of the subpopulation with an elevated MIC to FLC increased as a proportion of the total CSF fungal burden (the 2 other patients died before day 7). We next calculated the rate of change in %HetR per day as a summary statistic for FLC monotherapy versus combination treatment. In patients receiving FLC monotherapy, the rate of change of HetR was significantly greater than in those receiving the FLC/5FC combination (mean ± SD 4.55 ± 6.77 per day versus –0.70 ± 1.13 per day, respectively, *P* = 0.019). Amplification of HetR was higher in patients receiving the lower dose of FLC (800 mg/d) although this difference did not reach statistical significance (5.80 ± 7.78 per day versus 1.76 ± 2.62 per day, *P* = 0.19) (Figure 5B). Rate of change of HetR was not associated with baseline fungal burden (CFU/ml of CSF) or with the baseline HetR level (AUC of HetR in vitro) of the infecting strain.

In this small cohort, baseline HetR (as measured by AUC in the PAP assay) was not significantly associated with clinical markers of CM severity at presentation, including baseline fungal burden (*P* = 0.9), reduced Glasgow Coma Score (GCS) (*P* = 0.33), and raised intracranial pressure (*P* = 0.37), nor with 2-week, 10-week, or 1-year mortality (*P* values 0.5, 0.7, and 0.4, respectively).

### Combination therapy can prevent amplification of fluconazole heteroresistance in vitro.

We assessed whether this amplification or suppression of HetR could be replicated in vitro using the corresponding clinical strains. Two baseline (pretreatment) clinical strains were chosen: 1501, (from patient 15), in which HetR had amplified during FLC monotherapy, and 1901 (from patient 19), who had been treated successfully using combination FLC/5FC.

**Figure 6:**
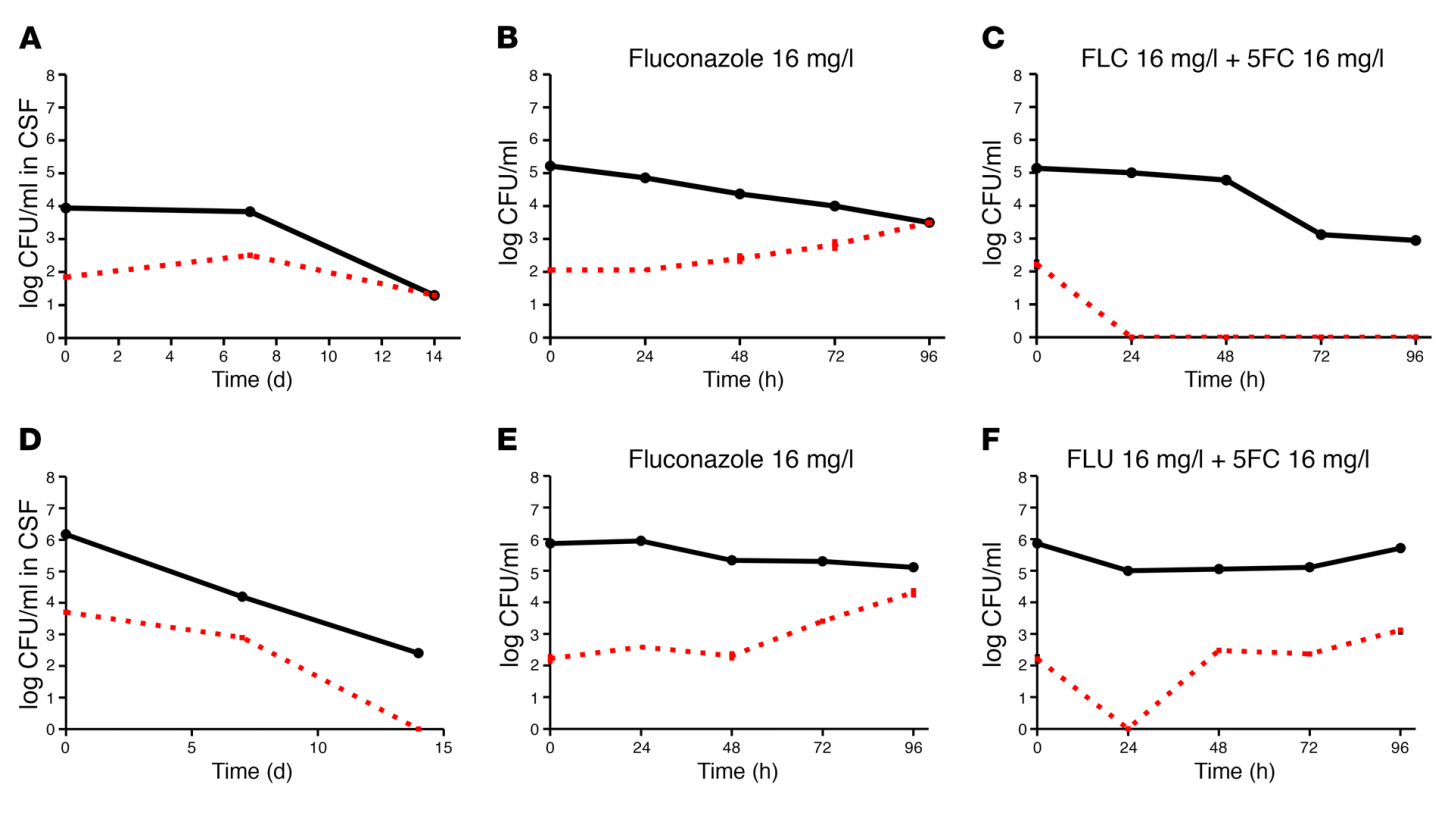
Amplification or suppression of heteroresistance in the clinical setting can be reproduced in vitro. In vitro experiments performed by inoculation of clinical strain into YPD with the addition of FLC with or without 5FC. Black line indicates total CFUs per milliliter at each sample time point. Red line indicates the heteroresistant subpopulation derived by CFUs per milliliter growing on YPD plus fluconazole. Data points with error bars represent the mean ± SEM of quadruplicated experiments. (**A**) Strain 1501 clinical data showing increased proportion of resistance. (**B**) Strain 1501 grown in YPD with the addition of 16 mg/l FLC. (**C**) Strain 1501 grown in YPD with the addition of 16 mg/l FLC and 16 mg/l 5FC. (**D**) Strain 1901 clinical data. (**E**) Strain 1901 grown in YPD with the addition of 16 mg/l FLU. (**F**) Strain 1901 grown in YPD with the addition of 16 mg/l FLC and 16 mg/l 5FC. This strain had acquired resistance to 5FC.

The 2 baseline (pretreatment) clinical isolates were maintained in liquid YPD plus 16 mg/l FLC, and YPD plus 16 mg/l FLC and 16 mg/l 5FC (also at 16 mg/l), conditions chosen to reflect plasma drug levels in the patients undergoing FLC monotherapy and FLC/5FC combination therapy, respectively (32, 33). The cultures were sampled daily for 5 consecutive days and plated to YPD alone and to YPD agar plus FLC (8, 16, 32, and 64 mg/l) and the total CFU and %HetR were calculated. For strain 15, %HetR increased during in vitro growth in YPD plus FLC, in a manner similar to that in the patient upon treatment (compare Figure 6A with Figure 6B), whereas when the same strain was grown in YPD plus FLC and 5FC medium, complete suppression of growth upon subculture on FLC agar was seen as early as 24 hours (Figure 6C). Similar results were obtained with strain 19. Exposure to FLC alone resulted in amplification of the HetR level in vitro (Figure 6E), and growth in FLC plus 5FC suppressed amplification of the HetR subpopulation on FLC similarly to that observed in vivo (compare Figure 6D with Figure 6F). However, in contrast to the clinical trajectories observed in the corresponding patient, in whom the subpopulation remained suppressed by combination therapy over time, during in vitro passage of strain 1901 with a combination of FLC and 5FC, HetR colonies reappeared 24 hours after initial suppression. Interestingly, at this point in the in vitro growth, strain 1901 progeny grown in vitro had acquired phenotypic resistance to 5FC (MIC by Etest of >32 mg/l), which effectively disabled the additional effect of the 5FC.

### HetR is associated with aneuploidy, particularly chromosome 1 disomy.

Cryptococcal DNA was extracted and sequenced from all 20 baseline isolates grown on YPD agar (i.e., from unselected colonies, strains 0101–2401) and from corresponding colonies from each of the 20 baseline strains grown on YPD with FLC (from 8–64 mg/l) added (HetR subpopulation, selected, strains 0101R–2401R). Additionally, where available, serial isolates (at the end of induction therapy and at clinical relapse if applicable) had DNA extracted for whole-genome sequencing (WGS). Strains sequenced are summarized in Table 1. DNA was extracted from single colonies. Sequence data are available at the European Nucleotide Archive (ENA, https://www.ebi.ac.uk/ena, accession number PRJEB28390). Sequences were analyzed for ploidy (based on genome-wide normalized coverage), and the presence of unique nonsynonymous SNPs between the parent and HetR isolates.

We recovered an average of 10.9 million reads from each isolate, with an average of 96.7% reads mapping to H99 (34), and an average of 59 times coverage (± 37 SD). High confidence variants were identified (see Methods) for further comparative and evolutionary studies. Full alignment, coverage, and variant calling statistics are provided in Supplemental Table 3.

Phylogenetic analysis revealed 19 *C*. *neoformans* isolates of the VNI genotype, and 1 *C. gattii* isolate of the VG1 genotype (isolates from patient 22). On average, 747 SNPs separated isolates within the same subclade, and isolates in this study accumulated on average 74 SNPs/day, ranging from 1 SNP/day/patient to 842 SNPs/day/patient. This wide range in SNP accumulation is likely to represent coinfection by genetically differentiated genotypes occurring in the environment. We found no nonsense mutations in either the *MSH2* gene or *SFG29* gene, which have been implicated as causes of hypermutators, that is, strains of *Cryptococcus* that accumulate large numbers of SNPs per day (35–37).

**Figure 7:**
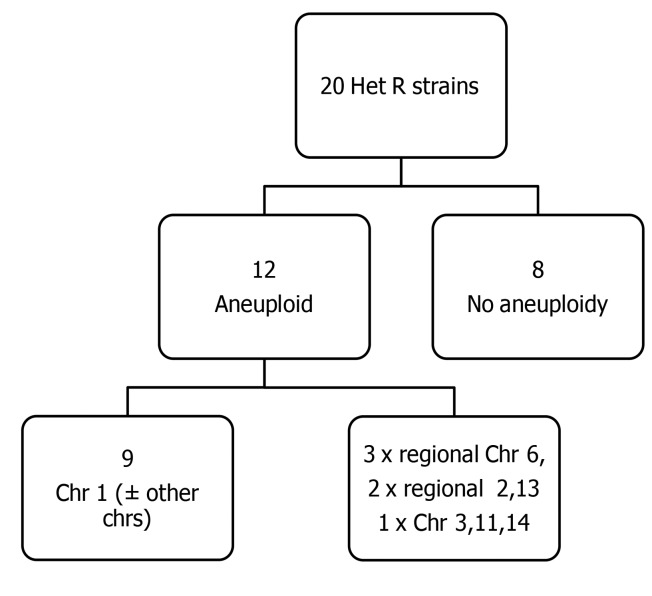
Whole-genome sequencing revealed high rates of aneuploidy in heteroresistant colonies. Summary of ploidy in resistant colonies compared with parental strains based on genome sequences and frequency of disomy per chromosome observed in the aneuploid clinical study isolates; chromosome 1 disomy was predominant.

**Figure 8:**
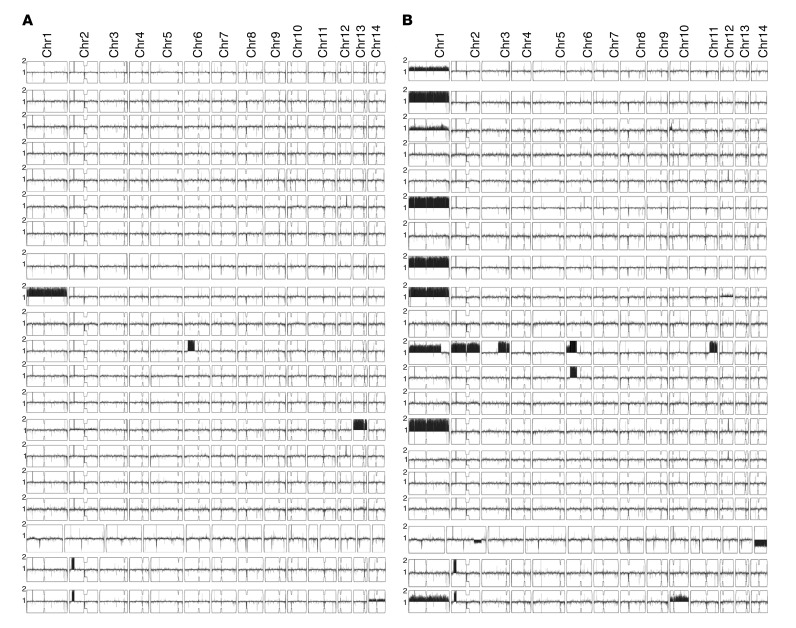
Aneuploidy is common in FLC-resistant colonies, in particular disomy of chromosome 1. Summary of ploidy data. (**A**) Chromosome maps of all 20 strains when a colony growing on plain YPD was sequenced. Only one strain has aneuploidy unselected colonies. (**B**) Corresponding chromosome maps of sequences from colonies selected on FLC-containing agar. Aneuploidy is common, in particular chromosome 1.

Aneuploidy of Chr1 was observed in a baseline unselected colony in 1 of the 20 isolates (strain 1101). Notably, this strain also had an elevated pretreatment FLC MIC (24 mg/l), and aneuploidy was present in the HetR colonies (selected by growth on FLC) in 12 of the 20 isolates (Figure 7). This was most commonly disomy of Chr1 (9 of 12 HetR baseline isolates), with partial or whole copy number variations of chromosomes 2, 6, 10, 11, 12, and 14 also observed in the HetR colonies. The ploidy of parental and HetR colonies of all 20 clinical strains are summarized in Figure 8.

In order to explore potential mechanisms of heteroresistance in the nonaneuploid strains, nonsynonymous SNPs unique to the HetR colony as compared with the unselected colony for all strains were determined from the sequence data. Nonsynonymous SNPs occurring in several coding genes were observed in more than one strain. Table 2 ranks these SNPs based on how frequently they were observed, and whether they were noted in aneuploid and nonaneuploid strains. Interestingly, nonsynonymous mutations of CNAG_03040, a transkelotase gene, were observed in 4 of the 8 resistant colonies of nonaneuploid strains, but not in any of the strains with chromosomal disomy. The significance of this is not immediately clear, but provides a candidate gene for further study.

### Clinical relapse and HetR is associated with Chr1 disomy.

**Figure 9:**
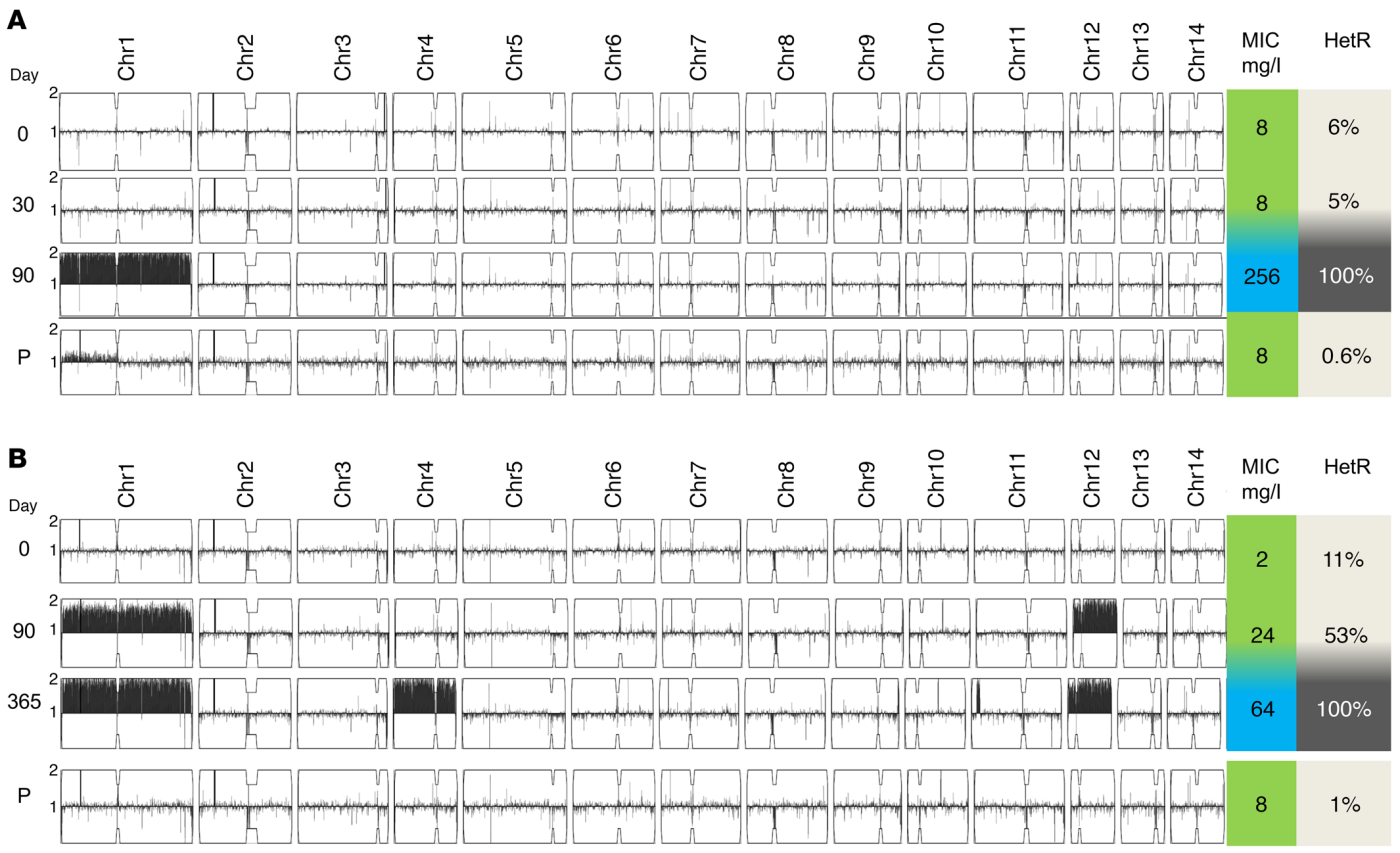
Clinical relapse of cryptococcal meningitis with high MIC to FLC is associated with transient chromosomal duplication. (**A**) Ploidy of sequenced (unselected) colonies from study patient 1 at baseline (pretreatment) and during FLU monotherapy (day 20 and at clinical relapse on day 90). The day 90 isolate was resistant (FLU MIC >256 μg/ml by Etest), with a corresponding increase in %HetR (proportion of colonies growing on fluconazole-containing agar as compared with total growing on drug-free YPD) and disomy of chromosome 1. The relapse isolate was then passaged serially for 12 days (labeled P) and then sequenced, revealing loss of disomy and reversion to a sensitive phenotype. (**B**) Another study patient (patient 2) who experienced 2 relapses at days 90 and 365, with a stepwise increase in disomies (1, 4, and 12) noted on isolates obtained from the patient CSF at each time point with corresponding increases in %HetR and MIC. As with the patient in **A**, on serial passage in plain YPD the isolate reverted to a euploid, low MIC phenotype.

Relapse of CM is a recognized risk of FLC monotherapy (12). In this study, 3 of the 15 patients on FLC monotherapy relapsed with symptomatic CM (headache, fever, altered mental status); none of the combination therapy patients relapsed. In all relapse cases, lumbar puncture was performed and *C*. *neoformans* was cultured from the CSF onto YPD (nonselective) media and sequenced. In 2 of the 3 patients, the relapse isolate was aneuploid and was associated with an increase in MIC compared with the original baseline parental strain, as well as an increase in %HetR at each relapse time point (Figure 9, A and B). In the third patient, in whom no changes in MIC or aneuploidy were observed, the relapse was considered to be due to the patient’s self-reported nonadherence to FLC.

To investigate whether the aneuploid chromosomes in these relapse isolates were stable, we cultured the relapse isolates for patients 1 and 2 in liquid YPD (without drug), and sampled them daily to measure MIC to FLC until reversion to baseline MIC was seen. Both relapse strains reverted to the baseline MIC after 10 and 12 passages, respectively. Importantly, reversion to the baseline MIC was accompanied by loss of the relevant aneuploidies (“P” in Figure 9, A and B), consistent with the hypothesis that the aneuploidies are responsible for the HetR phenotype in these isolates and that they incur a fitness cost in the absence of drug selection leading to a reversal of the HetR phenotype.

Of the patients who had CSF samples taken in the second week of induction treatment, sequences were obtained from colonies unselected by FLC agar, and examined for ploidy. Although amplification of HetR in terms of percentage of colonies growing on FLC-containing agar increased within this early period of treatment, we did not detect aneuploidy in unselected colonies at this early stage (11–17 days), unlike in the later relapse isolates described above. The only exception was strain 17, where disomy of Chr2 appeared at this early stage. The day 17 isolate had Chr13 disomy in the day 0 strain, yet Chr1 disomy was not detected at day 17 in the unselected culture, despite Chr1 disomy being present in the corresponding HetR colony at baseline (Figure 8). The diversity of genome sequence in this particular strain between parental and HetR colonies suggested a mixed population of *C*. *neoformans*, which may explain the diversity detected in different colony isolates.

### Heteroresistance is associated with increased efflux pump activity.

**Figure 10:**
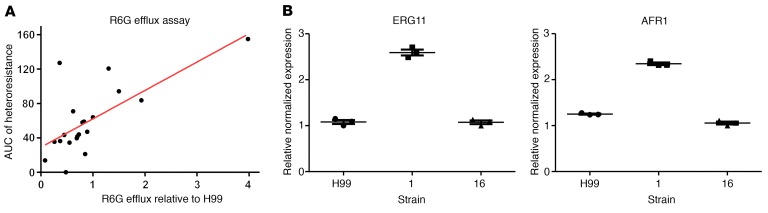
Heteroresistance is associated with increased efflux, and expression of ERG11 and AFR1 genes. (**A**) Heteroresistance is associate with increased efflux activity efflux activity (mean of triplicate experiments, normalized to H99, reference strain) correlates with heteroresistance as defined by AUC from PAP experiments (Pearson correlation coefficient *r* = 0.49, *P* < 0.001). (**B**) High efflux, disomic strain 1 compared with low efflux, euploid strain 16 shows approximately 2-fold increased expression of *ERG11* (fluconazole target) and *AFR1* (efflux pump). Data points represent results of experiments in triplicate with error bars displaying mean ± SEM.

The rhodamine 6G (R6G) assay measures the efflux activity of ABC-like transporters, which is ATP-dependent and is dramatically reduced in the absence of glucose (38). Glucose-induced efflux was measured in all 20 baseline strains and normalized to H99 efflux levels. Efflux of R6G correlated positively with the HetR AUC, with a lower, nonsignificant correlation with %HetR in patient CSF (Pearson *r* = 0.49, *P* < 0.001 and *r* = 0.15, *P =* 0.09, respectively, Figure 10A). In addition, there was a significant association between HetR isolates with Chr1 disomy and efflux activity (*P* = 0.004, Kruskal-Wallis test).

We next used RT-PCR to assay the expression levels of 2 relevant genes on Chr1: *AFR1,* which encodes a drug efflux pump, and *ERG11*, encoding the FLC target enzyme. A high efflux clinical strain with aneuploid heteroresistance (0101) and low efflux clinical strain with euploid heteroresistance (1601) were selected for comparison to the H99 reference strain. The genes *AFR1* and *EGR11* both exhibited significantly greater expression relative to the GPD gene (located on chromosome 7) in the Chr1 disomic HetR isolate 0101 as compared with strain 1601. The difference in expression was approximately 2-fold, presumably a consequence of disomy of Chr1 in strain 0101 as compared with the aneuploid 1601 (Figure 10B).

## Discussion

In this clinical microbiological study, we studied an African clinical cohort with intensive serial microbiological and genomic sampling of ex vivo patient CSF. We found that heteroresistance to FLC is intrinsic to clinical strains of *Cn* causing HIV-associated CM. In addition, CM induction treatment with FLU monotherapy can increase the size of HetR cryptococcal subpopulations that accumulate aneuploidies, a property associated with loss of microbiologic control and eventual clinical relapse with FLC resistance. Furthermore, this phenomenon appears to be suppressed by the use of FLC plus 5FC combination therapy in the clinical setting and in early time points of in vitro growth as well.

These results have clinical and microbiologic implications. In agreement with prior in vitro studies (24), most strains were heteroresistant at baseline, prior to the administration of any FLC treatment, in a strain-dependent manner. This implies that standing variation in the population of the infecting strain includes cells with aneuploidies that can provide a growth advantage in the presence of FLC. Routine diagnostic testing by Etest or broth microdilution does not measure HetR; it generally involves a subpopulation size that is too small to be detected with these assays. Thus, HetR is a subtlety missing in epidemiological MIC surveys that report low rates of primary resistance. Transience is a key feature of heteroresistance, distinguishing it from stable, genetic mutation-derived resistance. The transient nature of FLC resistance due to aneuploidy also confounds its detection because it is rapidly lost upon removal from drug pressure. PAP assays, the gold standard measure of HetR, are labor- and time-intensive and are unlikely to be suitable for routine clinical surveys. Novel techniques such as diskImageR, an image analysis pipeline for disk diffusion assays (39), can be useful for detecting tolerance and the consequent likelihood of recurrence of infection in *C*. *albicans* (28). However, *C*. *albicans* tolerance involves subpopulation sizes of greater than 10%. The low frequency of HetR in baseline populations may require the development of alternative approaches to measure HetR. With current clinical tools, heteroresistance is likely to continue to go undetected and to contribute to the failure of FLC monotherapy in treating human CM.

The combination of FLC with 5FC provides a faster rate of CSF fungal clearance as compared with FLC monotherapy (31). The ACTA trial, of which this is a substudy, recently reported the noninferiority of FLC plus 5FC combination therapy in terms of mortality outcomes compared with 2 weeks of AmB-based therapy, the long-standing gold standard, for induction treatment of HIV-related CM (40). Important advantages of the FLC plus 5FC combination are as follows: (a) it can be administered orally, (b) it is well tolerated compared with the relatively toxic AmB (29), and (c) it has the potential for generic manufacture, making it affordable for low and middle income countries (41). As a direct result of the ACTA trial, FLC plus 5FC as a combination is recommended in new WHO guidelines for induction treatment of CM in areas where AmB cannot be given (42). The data presented in our study provide evidence of a further benefit from combination therapy: it suppresses the amplification of HetR, an underestimated precursor of treatment failure, due to the appearance of resistance during the maintenance and consolidation phases of CM treatment, when FLC monotherapy is used as induction. Furthermore, persistence, tolerance, and heteroresistance are adaptive features across a wide range of microbial species, including eukaryotic and prokaryotic human pathogens (19, 43–45). Accordingly, we suggest that the principle of HetR suppression using relevant combination therapy strategies can be extrapolated to myriad other infectious diseases, including staphylococcal and mycobacterial infections.

The heteroresistance assay used here was performed ex vivo, that is, near bedside with minimal delay between sampling CSF from the patient and plating for cultures, and thus is a close representation of the phenotypic changes occurring within the human central nervous system during the course of cryptococcal infection. Genomic analysis of the isolates was performed after minimal passaging, although freeze/thaw cycles were unavoidably required at storage, shipment, and then receipt of samples. Analysis of isolates with as few passages as possible minimizes the pitfalls of repeated storage and passage that can lead to a loss of transient genomic changes such as aneuploidy, and have hampered prior studies of genomic evolution in serial clinical isolates of *C*. *neoformans* and *C*. *gattii* (46). Using this translational approach, we captured aneuploidy (most commonly disomy of Chr1) as a dominant, but not exclusive, feature of heteroresistant cells in the clinical setting. Relapse isolates of patients revealed stepwise duplication of whole chromosomes with an associated increase in MIC accompanied by clinical and microbiologic failure, in a manner similar to that observed in mouse studies (25). In addition, in one patient, an infecting strain with a high MIC to FLC was found to be disomic for Chr1 without any prior exposure to FLC. To our knowledge, this is the first such report of Chr1 disomy in a wild-type, baseline (untreated) infecting strain of *Cn* from a clinical case of CM.

We propose 2 mechanisms for the increase in the proportion of HetR cells within a population. First, selection during growth in FLC is expected to inhibit the growth of susceptible cells, allowing the preexisting HetR subpopulation to proliferate. Second, FLC has the ability to induce the appearance of loss of heterozygosity, polyploidy, and aneuploidy in both *C*. *albicans* (47) and *C. neoformans* (48). In *C*. *albicans,* FLC alters cell-cycle progression, resulting in the formation of trimeras, 3 connected cells whose aberrant nuclear replication ultimately results in unstable tetraploids that give rise to aneuploids (49). Similar effects of FLC exposure have been observed in *C*. *neoformans*, although no evidence was presented to show that aneuploid cells originate from multinucleated or polyploid cells (48). FLC induces the formation of multinucleated cells by causing uncoupling between nuclear division and cytokinesis. *Cn* has also been shown to form titan cells—large, polyploid cells that appear to have a significant impact on cryptococcal pathogenesis by evading phagocytosis by immune escape and host dissemination (50, 51). On exposure to FLC, titan cells appear to produce aneuploid daughter cells that are resistant to FLC (51). *Cn* titanization may play a role in generating the aneuploid subpopulations seen in *Cn*. Their role in promoting increased levels of heteroresistance has yet to be determined and is an avenue of future study.

Dysfunction in apoptosis regulation may also allow aneuploid cells to persist in the population. In the absence of *Aif1*, a protein that induces apoptosis-like cell death in *C*. *neoformans*, populations have a higher frequency of Chr1 disomy and FLC resistance (52). Nonetheless, no mutations of *Aif1*, unique to the HetR rather than unselected colonies, were found in the clinical isolates studied here.

The high incidence of aneuploidy and heteroresistance, even prior to exposure to FLC, suggests that aneuploidy is already present in environmental *Cryptococcus* strains, some of which will proceed to infect humans. Genome plasticity, and in particular aneuploidy, is increasingly recognized as an adaptive stress response in both bacteria and pathogenic eukaryotes (43). Indeed, aneuploidy has been noted in environmental strains of *Saccharomyces cervisiae* (53) as well as *C. neoformans* (54), although more rarely than in clinical isolates (55). Environmental strains of *C*. *neoformans* have also been found to be heteroresistant, suggesting many of these strains are likely to have been aneuploid, although ploidy was not determined in that particular study (24). Chromosomal copy number variation, in particular whole chromosome duplication (disomy in a haploid like *Cn*), is a potentially rapid mechanism to generate genomic diversity. Chromosome copy number increases may provide a selective advantage under many different stressors, including environmental challenges, due to the extra expression of hundreds to thousands of genes present on each chromosome (56).

The reversion to euploidy in the absence of drug stress suggests that aneuploids bear a fitness cost that is no longer worth bearing in the absence of fluconazole selection. A previous study found the heteroresistant colony derived from the H99 strain to be less virulent in mice than in the parental strain, and to grow more slowly in vitro (25). However, aneuploid strains appear, in this study, to retain sufficient virulence to cause clinical disease, either at baseline or relapse, and Sionov et al. in 2009 found highly HetR strains to be more virulent in mice than low HetR strains (24). This may be, in part, due to the profound immune suppression of the patients in our cohort, which can allow for relatively less fit strains to establish clinical disease.

Clearly, aneuploidy is not the only pathway to heteroresistance in *C*. *neoformans*, as in HetR clinical strains that were not aneuploid, the proportion of HetR colonies also increased during the course of therapy in the patient. The mechanism of heteroresistance in these nonaneuploid strains remains to be determined. Here we identified a number of candidate genes, based on recurrently observed SNPs in nonaneuploid yet heteroresistant and therefore transiently drug-resistant strains, that warrant further investigation (Table 2). Interestingly, nonsynonymous SNPs in *RAD57,* a protein involved in DNA repair, were seen in 6 of the strains, raising the possibility that this may have a role in the emergence of aneuploidy, although this will require further investigation.

This study is limited by small numbers and is therefore not powered to detect the significance of heteroresistance in clinical outcomes such as mortality. In addition, the method of detecting heteroresistance by culturing on FLC-containing agar raises the possibility of ex vivo induction of the phenomenon. However, it is unlikely induction would occur in the time course of a single passage. Efflux pump activity correlated with the HetR AUC derived from the PAP assays, though not with the %HetR colonies in the patients’ CSF at baseline. This is likely because PAP assays use a controlled inoculum and standardized methodology, in contrast to the bedside CSF analysis, which is subject to highly variable fungal burdens and host immune responses. The sequencing analyses are also limited by being based on sequences of single colonies, which does not account for the possibility of colony-to-colony variation or, indeed, the possibility of cell-to-cell variation. The advent of single-cell sequencing and the reducing cost of WGS may make sequencing of multiple colonies or single cells feasible in future studies.

In summary, this clinical study of the evolution and mechanisms of resistance to FLC in human CM is the first, to our knowledge, to detect the presence and emergence of FLC heteroresistance in human CM and to report the successful suppression of heteroresistance with 5FC plus FLC combination therapy. We believe this is the first prospective clinical study to examine the role of aneuploidy in CM patients, and to confirm and build upon previous work in a mouse model of *Cryptococcus* infection showing emergence of disomy during FLC treatment (23). In addition, this study suggests that the accumulation of aneuploidies, especially Chr1 disomy, is one of the mechanistic bases for clinical treatment failure and drug resistance. These findings provide another nail in the coffin of FLC monotherapy, which is an inadequate induction regimen for patients with HIV-related CM.

## Methods

### Observational clinical study.

From May 2015 to October 2016, nonpregnant patients older than age 18 with a laboratory-confirmed first episode of HIV-associated CM were recruited from Muhimbili, Amana, and Mwananyamala hospitals in Dar es Salaam, Tanzania. Prior to the start of the ACTA trial, patients received FLC treatment, as remains the local standard practice. After initiation of the ACTA trial, patients within the trial who were allocated to combination therapy with FLC at 1200 mg/day plus 5FC at 100 mg/kg/day (4 divided doses of 25 mg/kg/d) as induction therapy were approached for inclusion in the study. Lumbar punctures were performed on days 1, 7, and 14, or more frequently if clinically indicated. Patients were followed for 1 year.

### Ethics statement and study approvals.

The clinical study in Tanzania was performed with approval from the ethics committees of both the London School of Hygiene and Tropical Medicine (ethics reference 9176) and the National Institute of Medical Research in Tanzania (ethics reference NIMR/HQ/R.8a/Vol. IX/1905). Written informed consent was taken from all study patients or next of kin if they were unable to consent due to altered mental status.

### Strains used.

All clinical isolates were obtained prospectively from the clinical study performed for the purposes of this study and collected by the authors. Strains are labeled by patient number followed by the day of treatment; for example, strain 0101 is from patient number 1, day 1 of study (pretreatment). The C. *neoformans* var grubii H99 strain was used as a reference control strain in all in vitro experiments.

### Culture media.

Strains were cultured on yeast extract peptone dextrose (YPD) agar. Drug-containing plates were made by the addition of a stock solution of 50 mg/ml FLC powder (Alfa-Aesar) in DMSO, to final concentrations of 8, 16, 32, and 64 mg/l. Drug was added to the agar after autoclaving, then cooled to approximately 50°C before pouring. Penicillin/streptomycin (Hyclone, GE Life Sciences) was added to prevent bacterial contamination.

### Quantitative cryptococcal cultures.

These were performed using a modification of previously published protocol (57). Briefly, lumbar punctures were performed on patients on days 1, 7, and 14 of treatment if possible, or more frequently if clinically indicated. Samples were processed within 1 hour of collection from the patient. CSF was vortexed and plated onto YPD in serial 10-fold dilution for counting colony-forming units. CSF was also plated onto plain YPD agar and YPD with the addition of 8, 16, 32, 64, and 128 mg/l FLC. Plates were incubated at 30°C for 7 days and colonies were counted manually. CFUs per milliliter were calculated after adjustment for the dilution counted.

### MICs.

MICs to FLC were measured using the Etest method according to the manufacturer’s instructions (BioMérieux). Testing was done on-site in Dar es Salaam, Tanzania. A 0.5 McFarland standard suspension of the strain was prepared in 0.45% saline and spread in 3 planes using a cotton swab onto RPMI agar (Sigma-Aldrich). Etest strips (Biomerieux) were applied to the plate and incubated for 48 hours. MIC was determined as the point where the ellipse met the strip. MICs were performed in triplicate. The median was calculated and rounded up to the nearest concentration.

### Population analysis profile.

PAP assay was performed using a previously described method (17). Briefly, 10^6^ cells of *C*. *neoformans* were plated on plain YPD agar and YPD with 8, 16, 32, 64, and 128 mg/l FLC added to the agar. Serial 10-fold dilutions were plated in order to count colonies, and plates were incubated at 30°C for 5 days.

### In vitro drug evolution.

A 1 × 10^6^ suspension of cells in 10 ml YPD liquid (plus 1% penicillin/streptomycin) was prepared in 15-ml tubes for each experiment. FLC and 5FC were added to the tubes to a final concentration of 16 mg/l of both drugs within the tube. Tubes were incubated at 30°C and shaken at 225 rpm. Every 24 hours the tubes were removed and vortexed, and 1 ml of fluid was sampled. The sample was plated on YPD and YPD with FLC, and serially diluted 10-fold up to a 1 in 1000 dilution to facilitate colony counting. Plates were incubated at 30°C for 5 days and colonies manually counted, with CFUs per milliliter calculated after adjustment for the dilution factor of the plates counted.

### In vitro evolution of heteroresistance study.

From 1 colony of the study strain, a solution of 1 × 10^7^ cells in PBS was prepared. After vortexing, 1 ml of this suspension was added into 9 ml filtered YPD broth in 4 × 15 ml falcon tubes. Ampicillin was added to prevent bacterial contamination. Into each tube was added (a) nothing (control), (b) fluconazole to a final concentration of 16 mg/l, (c) flucytosine to a final concentration of 16 mg/l, and (d) both FLC and 5FC at 16 mg/l each. Each tube was vortexed and incubated at 30°C with shaking at 225 rpm. Every 24 hours, 1 ml was removed from each tube for quantitative cryptococcal cultures on both plain YPD and YPD with FLC added (as described earlier).

### R6G efflux assay.

Dynamics of R6G efflux were used to investigate ATP binding cassette (ABC) type drug transporter activity. After overnight growth to log phase in liquid YPD at 37°C, 10^9^ cells were inoculated into 20 ml fresh YPD and incubated at 30°C for 2 hours. Following centrifugation, the cell pellet was washed twice in PBS. Cells were resuspended in 10 ml of PBS. A quantity of 20 μM R6G (Sigma-Aldrich) was then added and the cells were vortexed before incubation at 30°C for 2 hours to allow cellular uptake of the dye. The cell pellet was then washed twice in PBS and resuspended in 5 ml fresh PBS. A quantity of 750 μl of this suspension was then aliquoted into microcentrifuge tubes, and 250 μl of either PBS or PBS with 8 mM glucose was added to each tube to make a final volume of 1 ml. Tubes were incubated at 35°C. After 30 minutes and then 1 hour incubation timepoints, tubes were removed and centrifuged at 805 *g* for 2 minutes. A quantity of 200 μl supernatant was transferred to a shallow 96-well plate and placed in an automatic plate reader (Synergy HT, BioTek). Fluorescence was measured at an excitation wavelength of 527 nm and an emission wavelength of 555 nm. Efflux in glucose-free supernatants was subtracted from that in glucose-containing supernatants to give an efflux value in relative fluorescence units (RFUs). *C*. *neoformans* H99 efflux was tested in each experiment, allowing efflux of each strain to be normalized to that of H99 and expressed as a ratio.

### RT-PCR.

Expression of the *AFR1* and *ERG11* genes was quantified by real-time reverse transcription PCR (RT-PCR). Yeast cells from strains H99, 0101, and 1601 were grown to logarithmic phase in YPD medium at 30°C with shaking. Each culture was then supplemented with 64 μg/ml FLC and allowed to incubate for 1 hour at 30°C with shaking. Cells were harvested by centrifugation, washed, and mechanically disrupted by vortexing (TissueLyser, Qiagen) with beads. Total RNA was then purified with the Qiagen RNeasy Mini Kit, including treatment with RNase-free DNase. cDNA was synthesized using the iScript Reverse Transcription Supermix (Bio-Rad). RT-PCR reactions were performed in triplicate using iTaq SYBR Green Supermix with the CFX96 Real-Time PCR Detection System (Bio-Rad). Data were normalized to the expression of glyceraldehyde-3-phosphate dehydrogenase (GPD). The *AFR1* primers used for RT-PCR were forward 5′-CCCACTTTGCCATACTTTTGG-3′, and reverse 5′-AACTGTGGAGACAAGACCACTGATAA-3′. The *Erg11* primers used were forward 5′-TGGCAAGACGCCAAAGTCT-3′, reverse 5′-GGCGGCAAATCCCTTTTC-3′.

### DNA extraction and sequencing.

Individual colonies (grown on YPD or YPD plus FLC) after a single passage from patient CSF were stored on Microbank beads (Pro-Lab Diagnostics) at –80°C. DNA extraction was performed on individual colonies after a single passage of the Microbank beads in pure culture onto YPD agar. Extraction method was with modification of the protocol for use of the Masterpure Yeast DNA Purification Kit (Epicentre), including a bead-beating step to disrupt the cryptococcal capsule (35). The bead beater (Tissuelyser LT, Qiagen) was used at 2 cycles of 1 minute each at 1/50 second. Quantification of DNA was performed by Qubit (Thermo Fisher Scientific). Shearing was performed using Covaris S220 (Covaris). The settings used were a 5% duty cycle, 200 cycles per burst, for 55 seconds, with the water bath cooled to below 8°C. For 100 ng gDNA in 54 μl volume in an Adaptive Focused Acoustics snap tube (Covaris), this gave a size profile with a median fragment size of approximately 400–450 bp. Quality control was performed using the Tapestation High Sensitivity D1000 ScreenTape (Agilent). DNA libraries were prepared using the NEBNext DNA library preparation kit (New England Biolabs). Sequencing was performed on the Illumina NextSeq platform with single-end reads of 150 bp length, at both the Weizmann Institute of Science, Rehovot, Israel, and the Centre for Genome Enabled Biology and Medicine at the University of Aberdeen, United Kingdom.

Raw Illumina reads were quality checked using FastQC v0.11.4 (Babraham Institute). All reads contained adaptor sequences which were trimmed using Trim Galore! v0.4.1 (Babraham Institute). All trimmed reads were aligned to the *C*. *neoformans* reference genome H99 (34) using the Burrows-Wheeler Aligner (BWA) mem v0.75a algorithm, and converted to sorted BAM format using SAMtools v1.2. Picard v1.72 (http://broadinstitute.github.io/picard) and Genome Analysis Toolkit (GATK) v3.4-46 were used to mark duplicates and preprocess the alignments prior to variant calling. Base recalibration was performed using GATK. Variants were called using GATK HaplotypeCaller and filtered to obtain a high-confidence variant that passed the parameters “DP < 5 || MQ < 40.0 || QD < 2.0 || FS > 60.0.” All SNPs were annotated using VCFannotator (Broad Institute).

Phylogenies of whole-genome SNP data were generated by RAxML v8.2.4 using the rapid bootstrap algorithm over 500 replicates for the best-scoring maximum likelihood tree. Phylogenies were visualized and annotated in FigTree v1.4.3 (http://tree.bio.ed.ac.uk/software/figtree/).

Depth of coverage was calculated using GATK from the whole-genome alignment. Ploidy analysis and chromosome coverage map figures were generated using the YMAP analysis pipeline (58).

### Statistics.

Graphs and AUC analyses were generated with Prism (v5, Graphpad Software Inc.). All other statistical analyses were performed using STATA version 13.1. A 2-tailed Student’s *t* test was used to compare mean changes in resistant subpopulations and total fungal between treatment groups. A *P* value of less than 0.05 was considered significant. Pearson correlation coefficient (*r* value) was used for the relationship between continuous variables, including AUC of heteroresistance and efflux of R6G, MIC, fungal burden in CSF, Glasgow Coma Score, and CSF opening pressure. The Kruskal-Wallis test was used for chromosomal disomy (categorical variable) and efflux (continuous variable).

### Study approval.

Studies in humans were reviewed and approved by the Observational/Interventions Research Ethics Committee, London School of Hygiene and Tropical Medicine, London, United Kingdom, and the Medical Research Coordinating Committee, National Institute of Medical Research, Dar es Salaam, United Republic of Tanzania. All study conduct was performed in line with Good Clinical Practice guidelines and the World Medical Association’s Declaration of Helsinki. Study subjects provided informed consent prior to their participation in the study. If study subjects were unable to provide informed consent due to their medical condition, informed consent was obtained on their behalf from their next of kin.

## Author contributions

NRHS, WH, and TB conceived the research question and clinical study with input from JKC. NRHS, SK, SFM, SM, and J Rugemalila conducted the clinical study, recruited patients, and collected clinical samples. TSH was the Principal Investigator of the ACTA trial, within which this study was embedded. NRHS conducted the onsite laboratory experiments and DNA extraction. NRHS and JB conceived the in vitro experiments. NRHS, ESS, and LN performed the in vitro experiments in JB’s laboratory. J Rhodes, NRHS, and MCF performed the sequence analysis. NRHS, JB, and TB wrote the manuscript, and all authors contributed to the final version of the manuscript.

## Supplementary Material

Supplemental data

## Figures and Tables

**Table 1:**
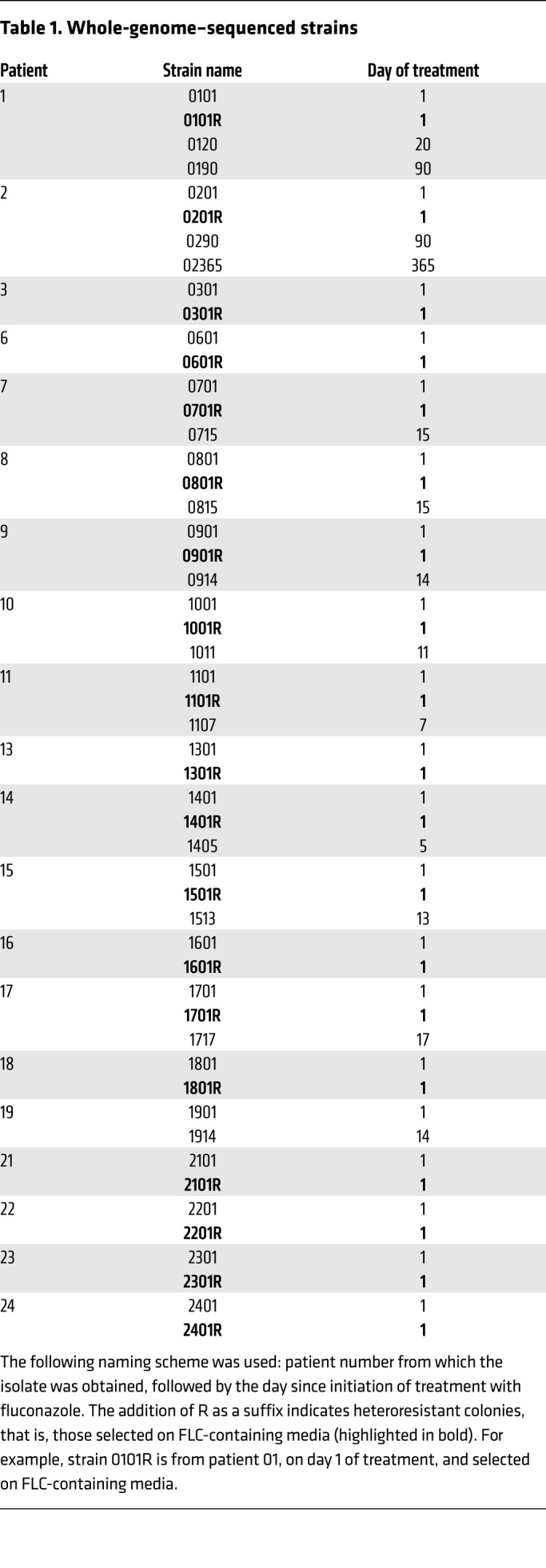
Whole-genome–sequenced strains

**Table 2:**
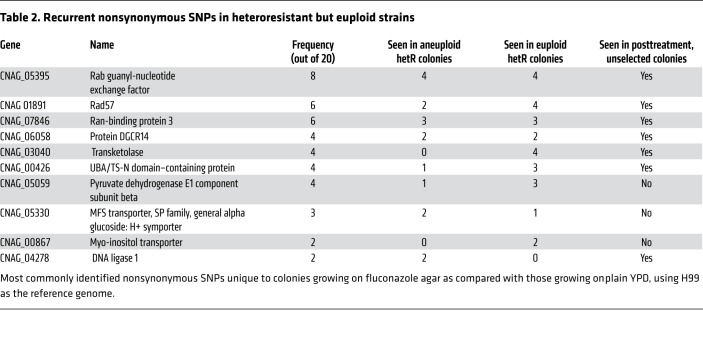
Recurrent nonsynonymous SNPs in heteroresistant but euploid strains

## Version 1 01/28/2019

Electronic publication

## Version 2 01/30/2019

Updated affiliation of Judith Berman.

## Version 3 03/01/2019

Print issue publication
