# Supplementary material for: Dynamic ploidy changes drive fluconazole resistance in human cryptococcal meningitis
Source: J Clin Invest. 2019 Jan 28;129(3):999–1014. doi: 10.1172/JCI124516 (PMC6391087; doi:10.1172/JCI124516)
Supplement: Supplemental data [file jci-129-124516-s034.pdf]

S1 Patients recruited – site and treatment received

PID	Site	Age	Sex	Fluconazole Dose
**001**	MNH	19	F	1200mg/day
**002**	MNH	35	M	1200mg/day
**003**	MNH	40	M	1200mg/day
**006**	MNH	50	F	1200mg/day
**007**	MNH	37	M	1200mg/day
**008**	ARH	33	F	800mg/day
**009**	ARH	40	M	800mg/day
**010**	ARH	48	F	800mg/day
**011**	ARH	42	M	800mg/day
**013**	ARH	40	F	800mg/day
**014**	ARH	31	F	800mg/day
**015**	ARH	50	F	800mg/day
**016**	ARH	30	F	800mg/day
**017**	ARH	45	F	800mg/day
**018**	ARH	40	F	800mg/day
**019**	MRH	43	M	5FC/FLU
**021**	MRH	30	M	5FC/FLU
**022**	ARH	29	F	5FC/FLU
**023**	MRH	33	M	5FC/FLU
**024**	MRH	53	M	5FC/FLU

PID -Patient identification number, MNH - Muhimbili National Hospital, ARH -Amana Regional Hospital, MRH- Mwananyamala Regional Hospital 5FC/FLU – patient received 1200mg Fluconazole + 100mg/kg/day 5FC

S2 Baseline characteristics of study patients

	n
**Total included**	20*
**Male**	9
**Female**	11
**Age (range)**	19-53
**CD4 cells/mm^3 median (IQR)**	35 (12-62)
**GCS < 15**	10 (50%)
**CSF Opening Pressure >20 cm H_20**	16 (80%)
**Baseline Fungal Burden CFU/ml CSF median (IQR)**	67,000 (10,100 – 902,500)
**Mortality**	
**2 weeks**	4 /20 (20%)
**10 weeks**	7/16 (44%)**
**1 year**	8/16 (50%)**

***24 recruited; 3 patients were culture negative and 1 isolate unusable due to contamination**

****4 patients lost to follow-up between 2 and 10 weeks**

S3 WGS coverage and SNP data

Isolate ID	# reads aligned (millions)	Depth of coverage (x)	% reference genome covered [a]	# filtered SNPs
**DAR0101**	62.0	207	99.0	44717
**DAR0101R**	5.4	36	98.6	44269
**DAR0201**	6.0	40	98.5	46437
**DAR0201R**	4.6	31	98.4	45012
**DAR0301**	11.0	68	98.6	46810
**DAR0301R**	8.5	52	98.5	46667
**DAR0601**	14.8	86	98.7	46876
**DAR0601R**	14.3	84	98.5	46861
**DAR0701**	4.0	28	98.5	44428
**DAR0701R**	16.8	94	98.1	46375
**DAR0801**	5.4	37	98.7	44006
**DAR0801R1**	6.9	45	98.6	44506
**DAR0901**	15.6	91	98.7	46918
**DAR0901R**	8.0	50	98.7	46542
**DAR1001**	6.0	41	98.3	44472
**DAR1001R**	6.6	42	98.5	44399
**DAR1101**	3.1	20	98.2	39423
**DAR1101R**	10.7	64	98.2	46648
**DAR1301**	6.4	42	98.7	46198
**DAR1301R**	7.1	45	98.2	46096
**DAR1401**	6.0	41	98.7	44490
**DAR1401R**	4.9	33	98.7	44244
**DAR1501**	56.9	198	98.5	46393
**DAR1501R**	4.8	33	98.5	43495
**DAR1601**	15.0	87	98.4	47558
**DAR1601R**	9.9	59	98.3	47142
**DAR1701**	4.8	32	98.7	43751
**DAR1701R**	13.9	83	98.4	46337
**DAR1801**	14.3	85	98.5	46349
**DAR1801R**	15.1	86	98.3	46360
**DAR1901**	4.8	33	98.5	46221
**DAR1901R**	13.7	83	99.3	47268
**DAR2101**	8.8	51	98.7	44176
**DAR2101R**	8.7	52	98.7	44250
**DAR2301**	15.4	86	98.6	46889
**DAR2301R**	14.3	83	99.3	47216
**DAR2401**	6.4	42	98.5	46700
**DAR2401R**	6.1	36	98.5	45542
